# Calciphylaxis Raising Suspicion for Factitious Dermatitis: A Diagnostic Challenge

**DOI:** 10.7759/cureus.93958

**Published:** 2025-10-06

**Authors:** Mónica F Santos, Tânia Alves, Diana Bernardo, Sandra Pereira, Palmira Coya

**Affiliations:** 1 Psychiatry, Unidade Local de Saúde de Santo António, Porto, PRT; 2 Liaison Psychiatry, Unidade Local de Saúde de Santo António, Porto, PRT; 3 Dermatology, Unidade Local de Saúde de Santo António, Porto, PRT; 4 Nephrology, Unidade Local de Saúde de Santo António, Porto, PRT

**Keywords:** calciphylaxis, cutaneous lesions, dermatitis artefacta, factitious dermatitis, psychodermatology

## Abstract

The term factitious dermatitis is often used interchangeably with dermatitis artefacta in the literature to describe self-inflicted skin injuries for which patients deny their responsibility. Its clinical presentation is diverse, and suspicion usually arises from clinical history and examination. However, it is crucial to exclude organic causes that may explain the lesions. We report the case of a young woman admitted for investigation of leg ulcers evolving over two months. Her medical history included renal disease since childhood, requiring long-term renal replacement therapy, as well as total thyroidectomy, hemiparathyroidectomy, acute alithiasic pancreatitis, secondary hypoparathyroidism, arterial hypertension, attention deficit, dyslexia, depression, and anxiety disorder. During hospitalization, the medical history and atypical presentation raised the suspicion of factitious dermatitis. Ultimately, the diagnosis of calciphylaxis was confirmed, a rare and life-threatening disease. In this case report, we stress the importance of a comprehensive assessment to rule out organic causes before establishing a primarily psychiatric or factitious diagnosis, while also emphasizing the value of a multidisciplinary approach in these cases.

## Introduction

According to the most recent version of the Diagnostic and Statistical Manual of Mental Disorders, dermatitis artefacta, also referred to as factitious dermatitis, is a term used in dermatology to refer to medically unexplained, self-induced skin lesions that the individual denies any role in creating. When there is evidence of deception, factitious disorder is diagnosed if the lesions arise in the absence of obvious external rewards (opposite to malingering) [1]. Similarly, according to the 11th revision of the International Classification of Diseases, artefactual skin disorder encompasses a diverse range of self-inflicted skin injuries that are provoked by the patient, whereas factitious disorder imposed on self is characterized by feigning, falsifying, or intentionally inducing or aggravating injury associated with identified deception, which is not solely motivated by obvious external rewards or incentives [2].

The term factitious dermatitis has been used interchangeably with dermatitis artefacta in the literature to describe self-inflicted skin injuries for which the patient denies responsibility. Its clinical presentation is varied, including excoriation, ulcers, blisters, crusting, eczema, purpura, and bruising as a few examples [3,4]. The suspicion of this condition can arise from clinical history and examination. Nevertheless, it is imperative to exclude organic causes that may be responsible for the lesions.

In this report, we describe the case of a young woman who presented with dermatological ulcers that raised suspicion of factitious dermatitis. Later, the diagnosis of calciphylaxis was confirmed, a severe vasculopathy characterized by calcium deposition in the arteriolar microvasculature of the deep dermis and subcutaneous adipose tissue [5]. It is classically associated with chronic kidney disease and carries a one-year mortality rate of up to 80% [5].

Through this clinical case study, we highlight the importance of ruling out organic causes for suspicious dermatological injuries, even when there is a suspicion of a primary psychiatric disorder.

## Case presentation

**Figure 1:**
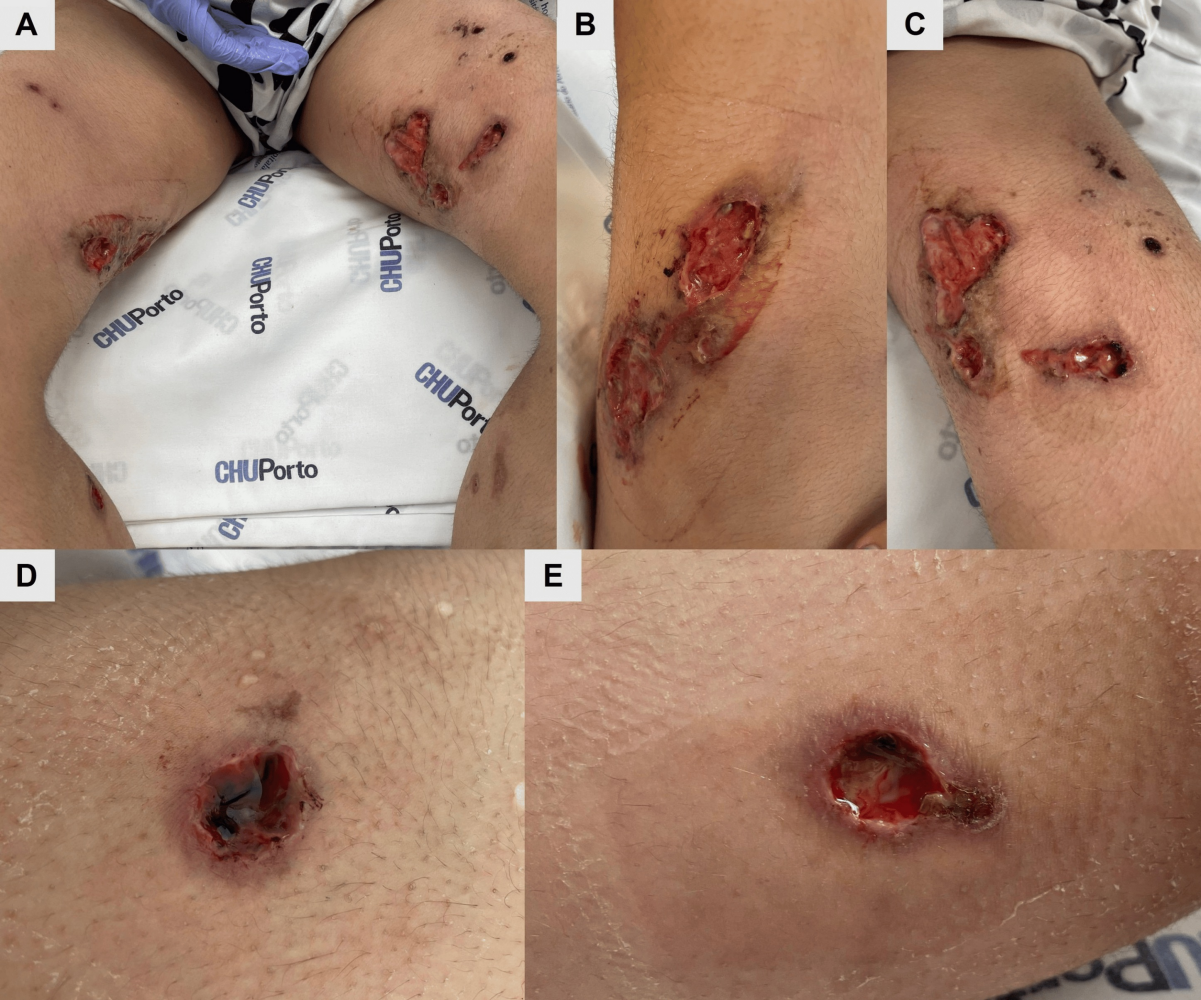
Images of the lesions at the time of admission. (A) Picture showing multiple lesions on the thighs of both lower limbs. (B) Oval-shaped lesion on the right thigh. (C) Irregularly bordered lesions on the left thigh. (D) Ulcer with violaceous borders and necrotic material. (E) Deep ulcer with hematopurulent exudate.

A 20-year-old woman was admitted for investigation of leg ulcers that had been evolving for two months. The lesions were initially managed in primary healthcare through debridement, wound care, and cefuroxime, without success. According to the patient’s description, they initially appeared as ecchymotic and vesicular, progressively evolving into necrotic ulcers with centrifugal growth. At the time of admission to the hospital, she presented several painful deep ulcers spread across both thighs, measuring 6 cm in the longest axis (Figure 1A-1C). Some were oval, confluent, others were irregularly shaped, with a “punched-out” appearance, violaceous border (Figure 1D), and base with foci of fibrin and hemorrhagic areas, accompanied by hematopurulent exudate (Figure 1E). A few irregularly shaped, hyperpigmented scars were also observed.

She had a medical history of corticosteroid-resistant nephrotic syndrome (IgM nephropathy), diagnosed at five years old, and stage five renal disease. She had initiated treatment with peritoneal dialysis at 15 years old and transitioned to hemodialysis at age 16. She underwent a renal transplant at 17 years old; however, it was complicated by chronic graft dysfunction. At 20 years old, three months before admission, she reinitiated hemodialysis. The adaptation to dialysis was poor, with sessions often being insufficient in duration. She also had poorly controlled secondary hypoparathyroidism. Her medical history further included autoimmune thyroiditis and follicular thyroid tumor requiring total thyroidectomy and hemiparathyroidectomy at 15 years old, acute alithiasic pancreatitis at 19 years old, as well as arterial hypertension. Regarding her psychiatric history, she had attention deficit and dyslexia diagnosed at eight years old, and a depressive episode and anxiety disorder diagnosed at 15 years old. She was medicated with sertraline 50-75 mg/day since age 16. Other medications at the time of admission were tacrolimus, prednisolone, erythropoietin beta, levothyroxine, alfacalcidol, and pantoprazole.

The laboratory analysis solicited during admission showed a hemoglobin level of 11.1 g/dL, elevated inflammatory markers (erythrocyte sedimentation rate of 64 mm/hour and C-reactive protein of 52 mg/L), elevated parathyroid hormone (326 pg/mL), and elevated serum phosphorus (1.54 mmol/L), with normal serum calcium levels (Table 1). There were no other significant abnormalities.

**Table 1:** Laboratory tests on admission.

Blood test	Values	Reference range
Red blood cells	3.96 × 10^6^/µL	3.8–4.8
Hemoglobin	11.1 g/dL	12–15
Hematocrit	37.8%	36–46
White blood cells	9.87 × 10^3^/µL	4.5–11
Platelets	368 × 10^3^/µL	150–400
Erythrocyte sedimentation rate	64 mm/hour	0–19
C-reactive protein	52.08 mg/L	0.0–5.0
Parathyroid hormone	326 pg/mL	15–65
Serum phosphate	1.54 mmol/L	0.87–1.45
Serum calcium (total)	2.44 mmol/L	2.15–2.50

After observation by Dermatology, a biopsy of the lesions was immediately performed and sent to the laboratory, and treatment with ciprofloxacin was initiated, as well as occlusive wound dressing and pain management with opioids. On the third day of hospitalization, observation by Liaison Psychiatry was requested, due to suspicion of factitious dermatitis. The suspicion arose in relation to the patient’s long medical history, the atypical presentation of the ulcers, and her extreme emotional reactions during wound dressing by the nursing team. During the psychiatric assessment, the patient described concisely and in detail the evolution of her condition and mentioned episodes of anxiety in relation to severe pain. She was collaborative, articulate, and had no other signs of psychopathological decompensation. The existence of tension between the nursing team and the patient was obvious, caused by the patient’s intense anguish during the dressing of the wounds and the suspicion that they were self-inflicted. The need for adequate pain management was reinforced, especially during wound debridement and dressing, as well as exclusion of a medical cause for the lesions through the histopathological examination. The assisting nursing team was also guided in the care and communication with the distressed patient. Diazepam 5 mg was prescribed as needed.

**Figure 2:**
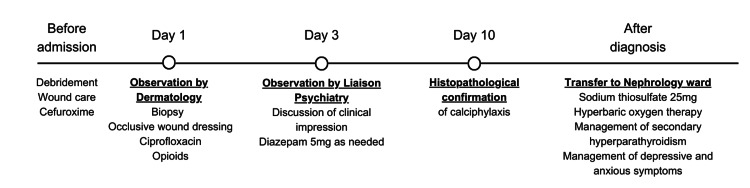
Treatment timeline.

On the 10th day of hospitalization, the histopathologic evaluation report was issued. A fragment of skin from the edge of an ulcer was analyzed, evidencing a lymphohistiocytic infiltrate, cystosteatonecrosis in the hypodermis, histiocytic inflammation, as well as small vessels and intralobular capillaries displaying extensive calcification of their walls. The result was, therefore, consistent with the diagnosis of calciphylaxis. The patient was then transferred to the Nephrology ward and successfully treated with sodium thiosulfate 25 mg after each hemodialysis session (three times a week), 22 sessions of hyperbaric oxygen therapy, and optimization of secondary hyperparathyroidism management, resulting in complete wound closure. She also initiated treatment with folic acid, amlodipine for arterial tension control, pregabalin for pain control after opioid suspension, and the sertraline dose was increased to 100 mg, as there was an aggravation of depressive and anxious symptoms reactive to her clinical situation. She was discharged 56 days after admission. The treatment timeline is shown in Figure 2.

## Discussion

The patient had a few characteristics that have been described in factitious dermatitis [3,4,6], including being female, single, with low income and lower educational level, and the age of onset of lesions reported in early adulthood. Moreover, she had a history of depression and anxiety disorder, which are frequent comorbid conditions [6], as well as a history of high utilization of healthcare services and a prolonged disease starting in childhood. As for the characteristics of the lesions typically associated with self-induced injury [3,4], she presented wounds with a punched-out appearance, located in areas accessible to the hands.

On the other hand, the ulcers, as well as the scars, were asymmetric and had irregular shapes, which is less consistent with the typical descriptions of self-induced lesions [3,4]. During evaluations, patients with factitious dermatitis tend to appear guarded, anxious, or even hostile while attempting to disguise their responsibility, and are usually not willing to describe the origin and first stages of the lesions or give information related to possible psychological stressors [6,7]. Some of them refuse a psychiatric evaluation [6]. This patient, however, described the history in detail, in a consistent manner, with a collaborative posture. Her emotional reactions were appropriate and congruent with the clinical situation. Moreover, another typical characteristic of self-induced skin lesions, in which patients appear to be able to predict the onset of new lesions that frequently emerge suddenly [4], was never reported. Although subjective sensations may be exaggerated by patients with factitious dermatitis [3], her complaints of excruciating pain appeared to be justified, considering the severity of the wounds. She also did not show a history or mental state examination consistent with obsessive-compulsive and related disorders or psychosis, dissociation, personality disorder, somatoform disorder, substance use disorder, or significant intellectual disability, more commonly associated with self-induced lesions [3,6,7]. Despite her previous diagnosis of depression and anxiety disorder, she did not have a history of non-suicidal self-injury. Moreover, she denied recent stressful events that could be contributing to psychological distress.

Although a high degree of suspicion is often crucial, none of the typical presentations is sufficient for the diagnosis of factitious dermatitis. Moreover, despite the possible suspicion, a confrontational and judgmental approach should never be used [6,8], as was reinforced in this case, while the nursing team was guided in communication with the patient. A thorough differential diagnosis is paramount, and the exclusion of the primary organic condition is essential.

In addition to the most common causes of skin lesions [8], less common conditions should also be considered. In this case, the histopathological examination was immediately requested, ultimately revealing the diagnosis of calciphylaxis. This disease is a vasculopathy that results from deposition of calcium in the arteriolar microvasculature of the deep dermis and subcutaneous adipose tissue [5]. It has been classically associated with chronic kidney disease, especially in patients treated with maintenance dialysis, who have developed secondary hyperparathyroidism, although serum calcium can be in the normal range [5]. One-year mortality rates range from 45% to 80%, and it is a rare disease [5], with few cases described at such a young age. This data reinforces the need for a thorough investigation and exclusion of all possible organic causes before diagnosing a self-induced lesion or factitious disease.

## Conclusions

In this report, we discuss the case of a rare and life-threatening condition that manifested through dermatological lesions with odd characteristics, leading to the diagnostic hypothesis of factitious dermatitis. We emphasize the need for a multidisciplinary approach in these cases and, above all, a thorough evaluation to exclude organic causes before concluding the presence of primarily psychiatric or factitious conditions. Additionally, although the index of suspicion for these cases may be high, we emphasize the importance of adopting an empathetic and non-confrontational stance, as failure to do so risks compromising appropriate management and treatment.
